# eGFR slope modelling predicts long-term clinical benefit with nefecon in a real-world IgAN population

**DOI:** 10.1093/ckj/sfae404

**Published:** 2024-12-14

**Authors:** Jonathan Barratt, Andrew M Stone, Heather N Reich, Richard A Lafayette

**Affiliations:** College of Life Sciences, University of Leicester, Leicester, UK; Stone Biostatistics Ltd, Crewe, UK; Department of Medicine, Division of Nephrology, University Health Network, University of Toronto, Toronto, Ontario, Canada; Division of Nephrology, Department of Medicine, Stanford University, Stanford, CA, USA

**Keywords:** end-stage kidney disease, glomerular filtration rate, IgA nephropathy, kidney failure, proteinuria

## Abstract

**Background:**

Nefecon is an oral, targeted-release formulation of budesonide approved to reduce kidney function loss in patients with immunoglobulin A nephropathy (IgAN). In the phase 3 NefIgArd trial, 9 months of nefecon treatment preserved estimated glomerular filtration rate (eGFR) and reduced urine protein–creatinine ratio versus placebo, for 15 months post-treatment. A modelling analysis was conducted to predict nefecon's long-term benefits on clinical outcomes.

**Methods:**

A published linear regression model was used to extrapolate nefecon's effect on the eGFR slope in NefIgArd to predict its effect on the clinical outcome of kidney failure, eGFR <15 ml/min/1.73 m^2^, or sustained doubling of serum creatinine. This model was applied to registry data from patients with IgAN at Leicester General Hospital (LGH), whose records were matched to individual NefIgArd patients on the basis of their urine protein–creatinine ratio and eGFR values.

**Results:**

A total of 1684 LGH-NeflgArd ‘matched pairs’ were obtained. Nefecon was predicted to delay the time to clinical outcome by 12.8 years (95% confidence interval 4.8–27.9), with median time to outcome of 9.6 years for patients receiving supportive care only versus 22.4 years for nefecon-treated patients. The NeflgArd 2-year eGFR slope yielded a log hazard ratio for the clinical outcome of 0.38 (95% confidence interval 0.21–0.63), a 62% risk reduction versus placebo. Of patients receiving only supportive care, 52% were modelled to have a clinical outcome within 10 years versus 24% of nefecon-treated patients.

**Conclusion:**

This modelling analysis indicates that the eGFR benefit seen with nefecon predicts a substantial delay in progression to kidney failure.

## Graphical Abstract

**Graphical Abstract:**
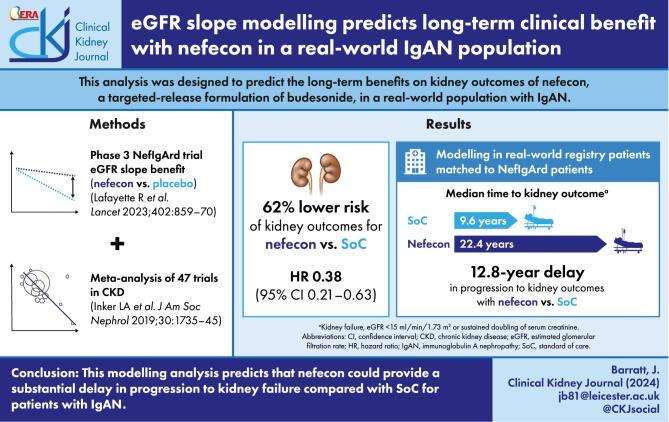

KEY LEARNING POINTS **What was known:**

- Immunoglobulin A nephropathy (IgAN) is an immune-mediated kidney disease characterized by a progressive decline in kidney function, which eventually leads to kidney failure in a large number of patients; a UK registry study showed up to 72% of adults progress to kidney failure within ∼20 years of diagnosis.
- An Inker *et al.* meta-analysis of clinical trials in patients with chronic kidney disease showed that treatment effects on 2-year estimated glomerular filtration rate (eGFR) total slope were predictive of reduction in the risk of a clinical endpoint of kidney failure, eGFR <15 ml/min/1.73 m^2^, or sustained doubling of serum creatinine.
- In the phase 3 NefIgArd trial, 9 months of treatment with nefecon, a targeted-release formulation of budesonide, was associated with reductions in proteinuria and preservation of eGFR versus placebo in patients with IgAN, for up to 15 months post-treatment.

**This study adds:**

- A linear regression model was used to extrapolate the effect of nefecon on eGFR slope in the NefIgArd trial to predict its effect on the clinical outcome of kidney failure, eGFR <15 ml/min/1.73 m^2^, or doubling of serum creatinine in a matched real-world cohort of patients with IgAN.
- The 2-year eGFR total slope reduction with nefecon was estimated to result in a 62% reduction in the risk of experiencing a clinical outcome compared with supportive care only.
- Based on modelling in a real-world UK population, the median time to clinical outcome was predicted to be delayed by 12.8 years with nefecon compared with supportive care only.

**Potential impact:**

- The results of this modelling analysis suggest that, by reducing the rate of kidney function loss, nefecon has the potential to delay the onset of clinical outcomes in patients with IgAN by many years.
- Furthermore, this analysis demonstrates the potential utility of modelling to extrapolate surrogate endpoint data from randomized controlled trials to predict treatment effects on clinical outcomes in a real-world setting.

## INTRODUCTION

Immunoglobulin A (IgA) nephropathy (IgAN) is a chronic immune-mediated disease of the kidneys [1, 2]. It is characterized by a progressive decline in kidney function attributed to the deposition of immune complexes containing galactose-deficient IgA1 (Gd-IgA1) in mesangial glomeruli [1, 3–5].

IgAN is associated with a very high lifetime risk of kidney failure [5–10], with up to 72% of adults in the UK National Registry of Rare Kidney Diseases (RaDaR) progressing to kidney failure within ∼20 years of diagnosis [5]. Kidney failure severely compromises patients’ quality of life, with poor long-term outcomes and high economic burden [11]. As kidney failure is defined by an estimated glomerular filtration rate (eGFR) <15 ml/min/1.73 m^2^, loss of eGFR over time is strongly associated with kidney failure [12–14]. Understanding how early reductions in eGFR in patients with IgAN might affect future development of kidney failure is important to inform discussions with patients and provide a greater understanding of the health and economic implications of using early interventions to delay kidney failure.

An eGFR decline of ≥30% is routinely used within definitions of kidney outcome endpoints in clinical trials, as a surrogate for progression to kidney failure [12–14]. Owing to the smaller populations and shorter trial durations in rare and progressive diseases such as IgAN relative to other kidney diseases, eGFR slope has been accepted as a practical surrogate for disease progression [14]. Shorter eGFR slope timeframes have been used to estimate clinical benefit, with a recent meta-analysis of 13 IgAN trials showing a 1-year eGFR slope to be a major, independent predictor of treatment effect on long-term clinical outcomes in IgAN [15]. A 2019 meta-analysis by Inker *et al*. assessed the use of eGFR slope as a surrogate endpoint for long-term kidney outcomes in 47 randomized clinical trials involving 60 620 patients with chronic kidney disease [16]. This analysis showed that treatment effects on 2-year eGFR total slope were predictive of a reduction in the risk of kidney outcomes, providing a robust model for predicting clinical outcomes from aggregated eGFR slope data [16]. Outside of the trial setting, validated tools such as the Kidney Failure Risk Equation and the International IgAN Prediction Tool can incorporate a wider range of individual patient variables alongside eGFR to predict disease progression [17, 18].

Evidence has shown that the mucosal immune system is involved in IgAN pathogenesis [2]. Within the mucosal-associated lymphoid tissue of the gut, Peyer's patches (aggregations of lymphoid follicles within the intestinal mucosal layer) act as major antigen and inductive sites, and evidence suggests that they are a key source of mucosal B cells that are primed to produce excess Gd-IgA1 in IgAN [2]. A therapy inhibiting Gd-IgA1 production in the gut-associated lymphoid tissue may be effective for primary IgAN [2, 19].

Nefecon (Calliditas Therapeutics) is an oral, targeted-release capsule formulation of budesonide, and is designed to act in the Peyer's patch–rich distal ileum to inhibit B-cell proliferation, class-switching, and maturation, thereby reducing excess Gd-IgA1 production [2, 4, 19, 20]. Nefecon became the first approved treatment by the US Food and Drug Administration for the reduction of kidney function loss in adults with primary IgAN at risk of rapid disease progression, based on the findings from the 2‑year phase 3 NeflgArd trial (NCT03643965) [21–24]. The trial demonstrated that 9 months of treatment with nefecon (16 mg/day) resulted in a statistically significant treatment benefit for the primary endpoint of time-weighted average change in eGFR over 2 years versus placebo of ∼5 ml/min/1.73 m^2^ (*P *< .0001) [2]. These results translated to ∼50% less kidney function deterioration with nefecon compared with placebo [2]. Additionally, nefecon exhibited a sustained effect on proteinuria, evidenced by a decrease in time-averaged urine protein–creatinine ratio (UPCR) of ∼41% versus placebo at 12‒24 months post-randomization (*P *< 0.0001) [2]. The composite clinical endpoint of a confirmed 30% reduction in eGFR or kidney failure was also significantly delayed with nefecon versus placebo (hazard ratio [HR] 0.45; *P *= .0014) [25].

In the current analysis, modelling was performed to estimate the time to the composite clinical endpoint of kidney failure, eGFR <15 ml/min/1.73 m^2^, or sustained doubling of serum creatinine (referred to as ‘the clinical outcome’) that would be predicted from the nefecon 16-mg treatment effect on 2-year eGFR total slope seen in the NefIgArd trial.

## MATERIALS AND METHODS

### Overview

Step 1: A published meta-analysis by Inker *et al*. modelled the relationship between eGFR slope and clinical outcomes [16]. This model was applied to the treatment effect of nefecon on 2-year eGFR total slope in the NefIgArd trial to estimate the expected effect on clinical outcome, quantified in terms of HR.

Step 2: Patients from the NeflgArd trial were matched to patients from the Leicester General Hospital (LGH) registry based on baseline UPCR and eGFR measurements, allowing for modelling of the treatment effect of nefecon in a real-world population receiving supportive care only.

Step 3: The time to clinical outcome (kidney failure, eGFR <15 ml/min/1.73 m^2^, or sustained doubling of serum creatinine) was estimated for the matched LGH reference group using a Weibull model. Using the HR estimated from Step 1, the time to clinical outcome was predicted for nefecon, as well as the proportion of patients estimated to have experienced an event within 10 years.

### Step 1: eGFR slope and relationship to clinical outcomes

The Inker *et al*. 2019 meta-analysis modelled the relationship between eGFR slope and kidney outcomes using a linear regression between treatment effects for the difference in 2-year eGFR total slope, analysed using a two-phase linear spline mixed-effect model, and the log HR of clinical outcome [16].

In the current analysis, a two-phase linear spline mixed-effect model was also used to estimate the difference in 2-year eGFR total slope in the NefIgArd trial between nefecon and placebo arms. Applying the linear regression coefficients of the Inker meta-analysis to the difference in 2-year eGFR total slope between groups in the NeflgArd trial, the estimated HR for the clinical outcome in NefIgArd was calculated. The 95% confidence intervals (CI) were calculated, allowing for the uncertainty in the NefIgArd treatment effect for 2-year eGFR total slope and the uncertainty in the relationship between 2-year eGFR total slope and HR of clinical outcome.

### Step 2: Matching to the Leicester General Hospital registry cohort

To quantify how the treatment effect of nefecon would translate to real-world outcomes, data were matched to a reference group from patients at LGH in the UK receiving supportive care only (renin–angiotensin system inhibitors, lifestyle modifications, and blood pressure control). The registry, initiated in 1992 at the John Walls Renal Unit, LGH, Leicester, UK, encompassed all patients diagnosed with primary IgAN via kidney biopsy, with ongoing data collection for these patients for the remainder of their life. The dataset used in this analysis was based on a data cut off of 1 January 2020. All samples were processed at the same laboratory at the University Hospitals of Leicester NHS trust, adhering to local clinical practice for assessment frequency. eGFR was estimated using the Modification of Diet in Renal Disease (MDRD) Study equation.

A unique patient ‘record’ was created for every date of recording of either a urine protein or eGFR measurement for an LGH patient, generating multiple unique ‘records’ for each LGH patient. As proteinuria and eGFR measurements were often not taken on the same day, the most recent proteinuria or eGFR value was carried forward for any given record with a missing value. An individual record was only included in this analysis if its eGFR and urine protein values met the criteria for eligibility in the NefIgArd study; that is, an eGFR ≥35–≤90 ml/min/1.73 m^2^, and proteinuria ≥1 g/day or UPCR ≥0.8 g/gram.

Each NefIgArd patient was matched to a maximum of five unique LGH records derived from different LGH registry patients. Matching occurred when the UPCR for an LGH record was within 25% and when eGFR was within 5 ml/min/1.73 m^2^ of the baseline value for a NefIgArd patient. NefIgArd patient eGFR values, originally calculated using the Chronic Kidney Disease Epidemiology Collaboration (CKD-EPI) equation, were converted to MDRD before matching.

**Figure 1:**
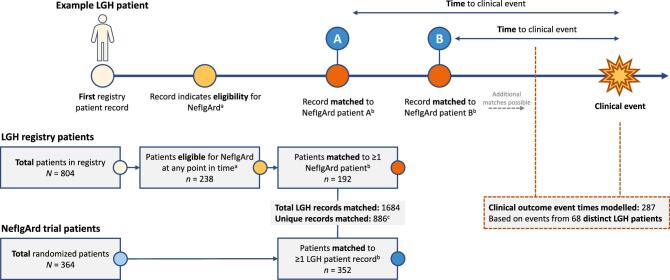
Cohort disposition. ^a^Based on UPCR and eGFR eligibility criteria. ^b^Different LGH patient records taken at different times were considered for matching. Each NefIgArd patient was matched to a maximum of five LGH records, from different individual LGH patients. LGH patients were matched if their UPCR was within 25% and eGFR within 5 ml/min/1.73 m^2^ of the baseline values for a NefIgArd patient. Therefore, multiple records from a single LGH patient could be matched to multiple NefIgArd patients. For 12 NeflgArd patients, there were no LGH records where both the UPCR was within 25% and eGFR was within 5 ml/min/1.73 m^2^ of their baseline values. ^c^If a unique LGH record was matched to multiple NefIgArd patients, its value was used only once in the modelling, resulting in 886 matched records being analysed.

If multiple records for the same LGH patient were identified as a match for the same NeflgArd patient (Fig. 1), the record with the closest match to the NefIgArd patient's baseline eGFR and urine protein values was chosen. If the same LGH patient record was matched to more than one NefIgArd patient, it was used only once in the modelling of LGH data. This provided a dataset of matched outcomes corresponding to patients included in the NefIgArd study.

When modelling the data, the time to a clinical outcome was calculated from the date of recording the matched LGH observation (Day 0). The endpoint modelled was the time it took for the earliest occurrence of kidney failure, eGFR decreasing to <15 ml/min/1.73 m^2^, or a doubling of serum creatinine from baseline. The serum creatinine and eGFR criteria required a second visit for confirmation, and the timing of this second visit was not restricted. It was not necessary for the second visit to immediately follow the initial event. Patients without an event were censored on their latest recording date.

### Step 3: Estimated time to clinical outcome

As described in Step 1, NefIgArd trial eGFR total slope data were used to calculate HR and associated 95% CI values for the predicted treatment effect of nefecon on the ‘clinical outcome’ (defined as the time to the earliest occurrence of kidney failure, eGFR <15 ml/min/1.73 m^2^, or sustained doubling of serum creatinine). This predicted treatment effect was applied to the matched LGH cohort by modelling LGH data using a Weibull distribution. To confirm the adequacy of the Weibull model, the resulting survival curve was superimposed onto a Kaplan–Meier estimate of the survival distribution of the same data.

The survival curve for nefecon and associated 95% CIs were also estimated using a Weibull model, with the same shape parameter estimated from modelling of the LGH data and multiplying the reference group scale parameter by the HR and corresponding 95% CI values for the HR.

The proportion of patients estimated to have an event within 10 years for the reference and nefecon 16-mg groups was also calculated using the same estimated survival curves.

## RESULTS

In this analysis, a total of 1684 LGH-NefIgArd ‘matched pairs’ were identified, stemming from 886 unique LGH records. Among these unique records, there were 287 distinct modelled clinical outcome events (kidney failure, confirmed eGFR <15 ml/min/1.73 m^2^, or confirmed doubling of serum creatinine). These records were derived from patients in the LGH registry: out of 804 patients listed, 238 patients had at least one record at a given point in time, which matched the eGFR and UPCR eligibility criteria for the NefIgArd trial. Among these, 192 patients had at least one record that was successfully matched with a NefIgArd patient based on matching UPCR and eGFR. There were 364 patients in the NeflgArd trial, of whom 352 were able to be matched with LGH patients in this analysis based on eGFR and UPCR values (Fig. 1).

Of all the patients in the LGH registry, 85% were of White ethnicity and 15% of East and South Asian origin, ∼65% were male, and no patients were treated with immunosuppression. Among censored LGH patients, median follow-up was 4.3 years, and maximum follow-up was 16.9 years. Baseline eGFR and UPCR values were similar between both NefIgArd trial and LGH registry matched records (Table 1).

**Table 1:** Cohort demographics: eGFR and UPCR summary statistics.

	NefIgArd patients (*n *= 352)	LGH records (*n *= 886)
eGFR (ml/min/1.73 m^2^), n	352	886
Mean (SD)	52.5 (13.1)	53.7 (13.1)
Median (IQR)	51.0 (42.5–62.0)	52.0 (43.0–62.0)
UPCR (g/gram), *n*	352	852^a^
Mean (SD)	1.47 (0.83)	1.64 (0.94)
Median	1.26	1.37

LGH eGFR values calculated using MDRD equation. NefIgArd eGFR values originally calculated using CKD-EPI, then converted to corresponding MDRD values before matching.

a In a small number of cases, if UPCR records were not available from LGH patients, matching was based on UACR.

UACR, urine albumin-to-creatinine ratio.

**Figure 2:**
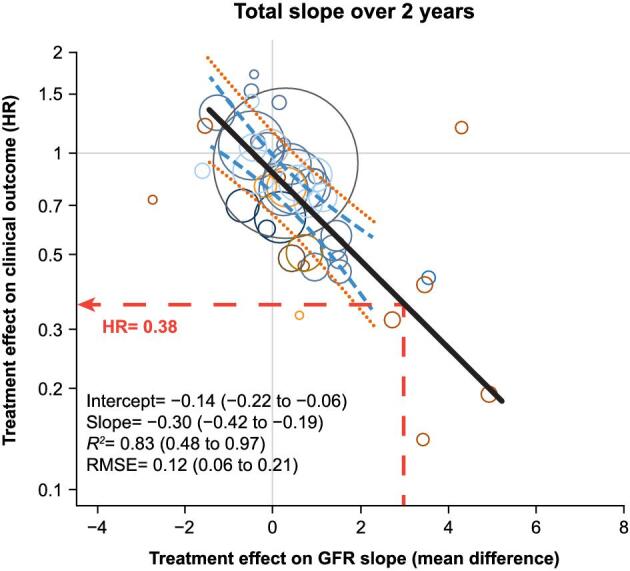
Relationship between treatment effects on 2-year eGFR slope and clinical outcome, with predicted HR for nefecon 16 mg. Adapted with permission from Wolters Kluwer Health, Inc.: Inker LA, Heerspink HJL, Tighiouart H *et al.* GFR slope as a surrogate end point for kidney disease progression in clinical trials: A meta-analysis of treatment effects of randomized controlled trials. J Am Soc Nephrol 2019;30(9):1735–45. https://journals.lww.com/jasn/abstract/2019/09000/gfr_slope_as_a_surrogate_end_point_for_kidney.18.aspx [16]. J Am Soc Nephrol is a journal of the American Society of Nephrology. The Creative Commons license does not apply to this content. Use of the material in any format is prohibited without written permission from the publisher, Wolters Kluwer Health, Inc. Please contact permissions@lww.com for further information. The meta-analysis of 47 trials in chronic kidney disease (Inker *et al*., supplementary data) [16] relating treatment effects on eGFR total slope to long-term clinical outcomes in IgAN was used to predict the HR associated with the treatment effect on 2-year eGFR total slope for nefecon 16 mg versus supportive care only in NefIgArd [21]. The black line represents the line of best fit between the treatment effects on 2-year eGFR slope and the clinical endpoint, which has been extended for presentation purposes. The blue lines are the associated 95% CI values for the mean effect, and the orange lines represent the 95% prediction intervals for outcomes from individual trials. A HR of 0.38 (95% CI 0.21–0.63) was predicted based on the NefIgArd analysis of 2-year eGFR slope, which showed a total eGFR slope benefit of 2.78 ml/min/1.73 m^2^ per year (95% CI 1.39–4.17) for nefecon 16 mg versus supportive care only [21]. The 95% CI values for the HR were calculated by allowing for the uncertainty in both the nefecon effect and the relationship between endpoints. RMSE, root mean square error.

In the NefIgArd trial, nefecon improved 2-year eGFR total slope by 2.78 ml/min/1.73 m^2^ per year (95% CI 1.39–4.17) compared with placebo when analysed using the 2-phase linear spline mixed-effect model. When this 2-year eGFR slope improvement was applied to the linear regression of Inker *et al*., an HR of 0.38 (95% CI 0.21–0.63) was predicted for the clinical outcome, in favour of nefecon (Fig. 2). This amounted to a 62% reduction in the risk of experiencing a clinical outcome with nefecon compared with placebo.

**Figure 3:**
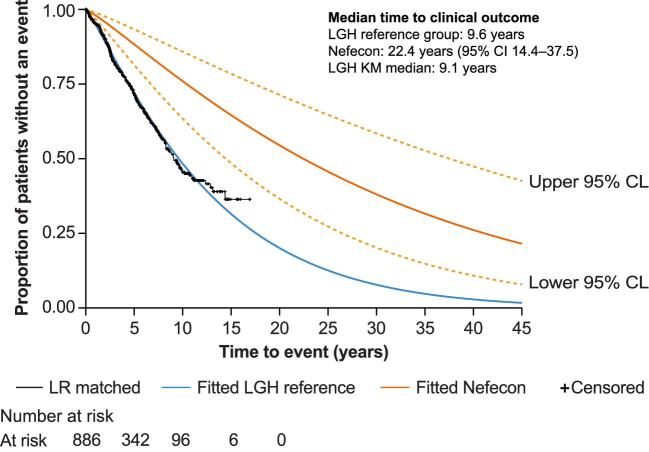
Time to clinical outcome estimated from LGH registry data and predicted outcomes for nefecon 16 mg based on the difference in 2-year eGFR slope in NefIgArd and Inker *et al*. meta-analysis. Individual patient records from the LGH registry, matched to NefIgArd patients based on baseline UPCR and eGFR, were used to estimate the median time to clinical outcome (kidney failure, eGFR <15 ml/min/1.73 m^2^, or sustained doubling of SCr) for a reference group receiving supportive care only (shown in black with censored time points). A parametric Weibull distribution was fitted to the data, censoring patients at their latest assessment, and estimated that the median time to clinical outcome was 9.6 years; this served as an estimate for the reference group (shown in blue). Conditional on this median, the HR (and 95% CI) estimated from the Inker *et al*. meta-analysis [16] was applied to predict the median time to clinical outcome for nefecon of 22.4 years (95% CI 14.4–37.5) (shown in orange). CL, confidence limit; KM, Kaplan–Meier; LR, linear regression; SCr, serum creatinine.

To estimate the predicted effect of nefecon on clinical outcomes in a real-world population, times to clinical outcome were calculated for the matched LGH reference group. Time to clinical outcome survival curves are shown in Fig. 3. The median time to clinical outcome was estimated at 9.6 years in patients receiving supportive care only and 22.4 years in patients receiving nefecon. This resulted in a delay in the median time to clinical outcome of 12.8 years (95% CI 4.8–27.9) with nefecon compared with supportive care only. In a *post hoc* sensitivity analysis, which assumed the effect of nefecon would reduce by 50% after 6 years, nefecon was predicted to delay the median time to clinical outcome by 7.1 years versus supportive care only.

Nefecon treatment benefit was also quantified in terms of the proportion of patients who would be expected to experience a clinical outcome event within 10 years. The associated 10-year event rate was 24% in patients treated with nefecon, compared with 52% in patients receiving supportive care only, a relative reduction of ∼50% with nefecon treatment.

## DISCUSSION

The long-term complications of kidney failure are considerable, both in terms of patient quality of life and economic burden [9, 11]. Therefore, delaying kidney failure is highly beneficial for patients, healthcare providers, and payers. In this analysis, the median time to clinical outcome was estimated to be 9.6 years for the reference group receiving supportive care only. Allowing for differences in definitions of clinical outcome and baseline proteinuria between studies, this was consistent with the RaDaR registry, as well as the Therapeutic Evaluation of Steroids in IgA Neuropathy Global Study (TESTING) and Supportive Versus Immunosuppressive Therapy for the Treatment of Progressive IgA Nephropathy (STOP-IgAN) trials [5, 26, 27]. In RaDaR, median time to kidney failure or death was 10.8 years in adults [5].

This modelling analysis predicts that a single 9-month course of nefecon treatment would delay the time to clinical outcome by a median of 12.8 years versus supportive care only (HR 0.38; 95% CI 0.21–0.63). This predicted treatment effect is consistent with the delay in the composite endpoint of time to confirmed 30% reduction in eGFR or kidney failure observed in the NefIgArd trial [2]. In the NefIgArd trial, clinical events were experienced by 21 (12%) of patients receiving nefecon versus 39 (21%) of patients receiving placebo, a significant 55% reduction in the risk of experiencing an event (HR 0.45; 95% CI 0.26–0.75) [2].

The observed treatment benefit of nefecon was also quantified to demonstrate an approximate 50% reduction over 10 years in the rate of clinical outcome events versus supportive care only. Therefore, even when restricting predictions over a time period closer to those reported in the Inker *et al*. meta-analysis [16], the observed effects of nefecon are predicted to translate into a clinically meaningful reduction in clinical outcomes.

It is noteworthy that this predicted long-term treatment effect is extrapolated from patients receiving a single 9-month treatment course of nefecon, as opposed to continuous treatment over 2 years. A benefit of this is that the 2-year eGFR slope therefore accounts for the loss of a direct treatment effect after the end of treatment, because it includes data from the 15-month follow-up period. However, there is uncertainty over the potential effects on long-term outcomes of nefecon treatment beyond 9 months, or of repeated treatment cycles. The NefIgArd open-label extension study showed that a second treatment course provides further kidney function benefit over a 9-month treatment period, similar to a first treatment course, supporting an additive treatment effect [28]. Additionally, preliminary results from a real-world study in US patients with IgAN showed continued preservation of eGFR with extended nefecon treatment beyond 9 months [29].

This analysis has primarily been focused on predicting the effect of nefecon on clinical outcomes based on its effect on eGFR, due to extensive evidence demonstrating that eGFR serves as an independent predictor of clinical outcomes [14–16, 19–27]. A meta-analysis of long-term clinical outcomes in IgAN showed 1-year eGFR slope to be a major, independent predictor of treatment effect. Interestingly, this same study found that, once the impact of 1-year eGFR had been considered, proteinuria reduction at 1 year or earlier was not found to be an independent predictor of clinical outcomes [15]. Nonetheless, a meta-analysis by Thompson *et al*. [30] established a ‘reasonably likely’ relationship between proteinuria and clinical outcomes. Using the regression determined by that analysis, the 50% reduction in UPCR at 12 months observed in the NefIgArd trial would provide a 67% reduced hazard of kidney failure or death (HR 0.33; 95% CI 0.16–0.67) [30, 31]. Consistent with the estimates based on eGFR slope, it is predicted that the effect of nefecon on proteinuria would translate into a similar delay in the median time to clinical outcome [30, 31].

By applying insights from published clinical studies and integrating them with real-world data, this analysis effectively addresses the challenges posed by the infrequency of kidney failure events in clinical trials focused on IgAN, which often span only a few years. However, this analysis does have a number of limitations. First, the LGH registry data were retrospective and the treatment of patients in this time period did not reflect the latest draft 2024 KDIGO (Kidney Disease: Improving Global Outcomes) guidelines, which include the use of sodium-glucose cotransporter-2 inhibitors and dual endothelin angiotensin receptor antagonists [32]. Moreover, the use of single-centre data imposes specific limitations; for example, LGH is an Academic Centre with a particular focus on IgAN management, where care practices may more closely align with those of a clinical trial than the broader healthcare system. Other sites may be less strict in ensuring that all patients are on maximum tolerated doses of renin–angiotensin system inhibitors and that blood pressure is maintained <125/80 mmHg. Additionally, LGH patients were predominantly White and are not representative of the global IgAN population. Although data from the NefIgArd trial do indicate nefecon had a similar treatment effect in White and Asian patients [2, 33], caution should nonetheless be exercised when extrapolating the findings of this analysis to East and South Asian populations with IgAN.

The two populations, from the NefIgArd trial and LGH registry, were not specifically selected for the purpose of this analysis; however, the strict matching criteria applied at an individual patient level are expected to mitigate any differences between the two populations. Because of these strict matching criteria, the number of registry patients included in the analysis was relatively small, although this precise individual matching is also a strength, allowing for robust data extrapolation from the trial to the real world.

Last, it is important to acknowledge that the model's predictions rely on the assumption that HRs remain accurate with further follow-up. The median follow-up for clinical endpoints in the studies included in the Inker *et al*. meta-analysis was ∼3 years, ranging from 1.4 to 6.5 years [16]. Of note, the pooled analysis of IgAN steroid studies had among the longest median follow-up periods in the meta-analysis (6.5 years) and did not show any evidence of a diminished treatment effect [16]. This suggests that HRs estimated from eGFR data remain accurate for at least 6–7 years. Additionally, the treatment benefit of nefecon on clinical outcomes and UPCR in NefIgArd suggests it may provide longer-lasting kidney protection. Nevertheless, a sensitivity analysis was conducted, predicting that, even if the effect of nefecon was assumed to reduce by 50% after 6 years, nefecon would delay the median time to clinical outcome by 7.1 years versus supportive care only.

Collectively, these modelling analysis findings, along with the positive impact seen on individual clinical endpoints in the full 2-year NefIgArd trial data, suggest that nefecon, the first therapy to be proved to reduce IgA immune complex formation in IgAN [34], has the potential to delay the onset of clinical outcomes in patients with IgAN by many years.

## Data Availability

Data sharing requests will be considered from research groups that submit a research proposal with a valuable research question and an appropriate statistical analysis and dissemination plan. Proposals will be assessed by a committee formed from the trial management group, including senior statistical and clinical representation. Data will be shared via a secure data access system.
